# Discriminant Input Processing Scheme for Self-Assisted Intelligent Healthcare Systems

**DOI:** 10.3390/bioengineering11070715

**Published:** 2024-07-14

**Authors:** Mohamed Medani, Shtwai Alsubai, Hong Min, Ashit Kumar Dutta, Mohd Anjum

**Affiliations:** 1 Applied College of Mahail Aseer, King Khalid University, Abha 62529, Saudi Arabia; 2 Department of Computer Science, College of Computer Engineering and Sciences in Al-Kharj, Prince Sattam bin Abdulaziz University, P.O. Box 151, Al-Kharj 16278, Saudi Arabia; 3 School of Computing, Gachon University, Seongnam 13120, Republic of Korea; 4 Department of Computer Science and Information Systems, College of Applied Sciences, AlMaarefa University, Ad Diriyah, Riyadh 13713, Saudi Arabia; 5 Department of Computer Engineering, Aligarh Muslim University, Aligarh 202002, India; mohdanjum@zhcet.ac.in

**Keywords:** emotion data, intelligent computing, transfer learning, healthcare system

## Abstract

Modern technology and analysis of emotions play a crucial role in enabling intelligent healthcare systems to provide diagnostics and self-assistance services based on observation. However, precise data predictions and computational models are critical for these systems to perform their jobs effectively. Traditionally, healthcare monitoring has been the primary emphasis. However, there were a couple of negatives, including the pattern feature generating the method’s scalability and reliability, which was tested with different data sources. This paper delves into the Discriminant Input Processing Scheme (DIPS), a crucial instrument for resolving challenges. Data-segmentation-based complex processing techniques allow DIPS to merge many emotion analysis streams. The DIPS recommendation engine uses segmented data characteristics to sift through inputs from the emotion stream for patterns. The recommendation is more accurate and flexible since DIPS uses transfer learning to identify similar data across different streams. With transfer learning, this study can be sure that the previous recommendations and data properties will be available in future data streams, making the most of them. Data utilization ratio, approximation, accuracy, and false rate are some of the metrics used to assess the effectiveness of the advised approach. Self-assisted intelligent healthcare systems that use emotion-based analysis and state-of-the-art technology are crucial when managing healthcare. This study improves healthcare management’s accuracy and efficiency using computational models like DIPS to guarantee accurate data forecasts and recommendations.

## 1. Introduction

Modern society heavily relies on clever systems. Anytime an individual uses an intelligent system, the network or process provides with better services and runs more efficiently. Emotionally grounded intelligent systems are a relatively new field of study. Feelings are inherent to all living things, including humans [1]. Regarding intelligent systems’ interaction and analysis, emotions are crucial. It is possible to record people’s facial expressions using surveillance cameras. The classification of human facial expressions under specific situations allows for the development emotion-based intelligent systems [2]. Integrating emotion-based analysis with modern technology, self-assisted intelligent healthcare systems improve services by offering observation-based self-assistance and diagnoses; these systems are then used in healthcare institutions. These systems help with diagnosis, treatment planning, and patient care by analyzing patient data, including emotional data, then making individualized suggestions and improving the accuracy of healthcare administration. One way to classify emotions is with the use of a fuzzy means algorithm that is based on deep learning.

In the field of mental health, electroencephalograms (EEGs) have the potential to aid in diagnosis and fast feedback on treatment strategies for mental health problems, such as depression, deteriorating health due to certain behaviors, etc. The electrical activity of brain cells can be detected by EEGs, which are then used to extract features. The use of microphones in healthcare settings allows for the detection of patients’ emotional states through the analysis of their speech signals and body language. We may examine emotional states using AI markup language by observing neural activity. Electronic brain waves (EEGs) are used in healthcare management systems to assess patients’ emotional states. According to studies, an individual’s emotional state can be deduced from electroencephalogram (EEG) data collected from facial expressions and actions. Improvements in patient treatment are possible, thanks to EEG’s higher performance compared with other analytic methods, which provide information regarding a patient’s mental health and thoughts.

Clustering techniques are used to change the expression differences from the provided data. Emotional intelligence apps are primarily utilized in e-healthcare systems to enhance service quality [3]. The Sensitive Artificial Listener framework is used to analyze people’s emotions. The primary goal of this framework is to improve communication within the company by keeping an eye on employee expressions [4]. Smartphones employ a convolutional model as part of their emotion recognition mechanism. Such a method helps understand user sentiment, which improves desired service quality and boosts overall performance [5]. 

There is extensive use of emotion-based intelligent systems in healthcare applications and systems. The analysis process and the provision of better, timelier services to patients are improved by healthcare applications that use emotions. Electronic health records use electroencephalogram (EEG) signals to determine a patient’s emotional state [6]. EEG is a tool for diagnosing mental health issues and providing timely feedback for individualized treatment plans. In mental health issues, including depression, worsening health as a result of activities, etc. [7], EEGs detect the electrical activity of brain cells that are subsequently used to extract characteristics. In healthcare applications, microphones can detect patients’ emotions based on their vocal signals and movements [8].

Affect Aura can determine patients’ moods using their visual, auditory, mental, and other gathered data. Predictions in healthcare applications are the most commonly utilized. Emotions are classified using pre-existing case data and pre-existing category labels [9]. The healthcare system improves its services to impacted patients using state-of-the-art algorithms [10]. Advanced models are utilized to predict an individual’s emotions based on their behavior, enhancing emotion identification accuracy [11]. Every system and management heavily relies on emotion analysis. The k-nearest neighbor technique, support vector machines, and quadratic discriminant analysis are among the analysis processes. Artificial intelligence markup language is utilized to analyze emotions based on brain activity [12]. One tool in healthcare management systems for examining patients’ emotions is the EEG. According to research [13], EEG data from patients’ expressions and activities can be used to analyze their emotional state. By providing insight into a patient’s mental health and thoughts, EEG delivers superior performance compared with other analytic processes, improving patient treatment. The expressed emotions of the patient and their loved ones are analyzed using the Expressed Emotions approach [14]. Using data gathered from surveillance cameras, it deduces people’s facial expressions and emotional states. Robots are increasingly finding applications in healthcare, particularly in analyzing patients’ emotions and activities [15]. Healthcare monitoring is the main focus of the conventional approach. Regardless, there are a few drawbacks, such as the reliability and scalability of the fractal pattern feature generation method concerning various EEG data sources and neurological conditions—more evidence of the efficacy of the emotion analysis paradigm in real-world clinical contexts and with different demographics. Complex nonlinear systems frequently give particular challenges when modelled on a global scale. First, the complexity of data distribution patterns makes it hard to estimate them, making it much more difficult to ascertain the model’s structure. Process-monitoring models trained using the unimodal assumption could generate many false alarms.

A patient’s emotional state can be deduced from facial expressions, mental state, and auditory or physical symptoms [16]. The main objective points are as follows:

- To improve self-assistance services and diagnoses through emotion-based analysis and advanced technology in healthcare data.
- To assess emotional data in intelligent healthcare systems, the study has introduced DIPS, which uses cutting-edge processing methods.
- To enhance the accuracy of DIPS’s recommendations by identifying similar data patterns across multiple streams.

The following is a summary of the research that was conducted. A comprehensive analysis of the existing research methods and literature is carried out in the second section. The processes for processing, the research methodology, and the research plan are all described in Section 3. A discussion of the analysis of the result is found in Section 4. In the fifth section, the primary conclusion and future work are discussed.

## 2. Related Works

In Table 1, the survey finds healthcare monitoring, emotion identification, wise ageing, wound healing, and smart city traffic management research deficiencies. These gaps include integrating emotion recognition systems into healthcare monitoring, optimizing mobile healthcare app data management, improving emotion analysis frameworks, exploring biomedical applications of hydrogels, improving smart ageing solutions, and integrating real-time emotion recognition data in traffic management. More research is needed to improve health treatment, data preservation, and older adults’ quality of life.

Li et al. [19] present a multistep deep (MSD) approach that can accurately identify multimodal emotions from records that may contain inaccurate information. Additionally, the MSD system uses specialized deep neural networks to extract features from visual and physiological inputs, taking spatiotemporal information into account. Using an open-source multimodal database for the simulation trials, we find that the MSD system outperforms the conventional one regarding unweighted average recall. Positive experimental results confirm the suggested system’s potential impact on real-world IoT applications.

Tuncer et al. [20] present the fractal Firat pattern (FFP), which takes its name from the logo of Firat University. This paper introduces a multilayer feature generator using FFP and TQWT signal decomposition techniques. An approach to emotion recognition based on fractal pattern features was suggested in the article (FPFEA). Iterative selectors were used in the feature selection process. This model was evaluated on 14-channel emotional EEG signals using a support vector machine (SVM), k-nearest neighborhood (k-NN), and linear discriminant analysis (LDA). The suggested framework’s SVM classifier reached 99.82% accuracy.

Pane et al. [27] provide the results of an EEG-based public emotion dataset that includes four categories: happy, sad, furious, and relaxed. The most effective classifier in ensemble learning is valence lateralization random forest (VL-RF). Using grid search optimization, the RF model’s parameters were fine-tuned. We used SVM and LDA, two popular EEG methods, to compare RF. By utilizing three pairs of asymmetry channels—T7–T8, C3–C4, and O1–O2—the accuracy of emotion classification improved dramatically compared with the absence of lateralization. Among the three methods tested, RF achieved the best classification accuracy (75.6% vs. 69.8% for SVM and 60.4% for LDA).

Gong et al. [30] present a new model for EEG emotion identification that considers the brain’s hemispheric asymmetry along with the dimensional, wavelength, and spatial multi-domain characteristics of EEG signals. The goal of this model is to enhance the effectiveness of emotion detection. The two matrix models train a convolutional neural network that can extract depth characteristics in two different streams: spatial and spectral. We achieved stellar average results of 98.33%/2.46%, 92.15%/5.13%, 97.50%/1.68% (valence), and 97.58%/1.42% (arousal) on the SEED, SEED-IV, and DEAP public datasets, respectively, during our comprehensive trials.

Kamble et al. [31] suggest a noise-free method for obtaining the desired EEG frequency range for emotional emotion identification using a dual-stage correlation and instantaneous frequency (CIF) threshold. There is evidence from this study that EML classifiers outperform CML classifiers, specifically in an ensemble setting. The most effective F1 scores were 84.53% for arousal, 76.24% for valence, and 89.16% for dominance, as reported by random forest with differential entropy features. Compared with the three CML classifiers, the average F1 scores of the three EML classifiers were around 2.30%, 7.49%, and 2.65% higher, respectively. Finally, the proposed CIF-based filtering approach aids EML classifiers in detecting emotional states.

## 3. Discriminant Input Processing Scheme

**Figure 1:**
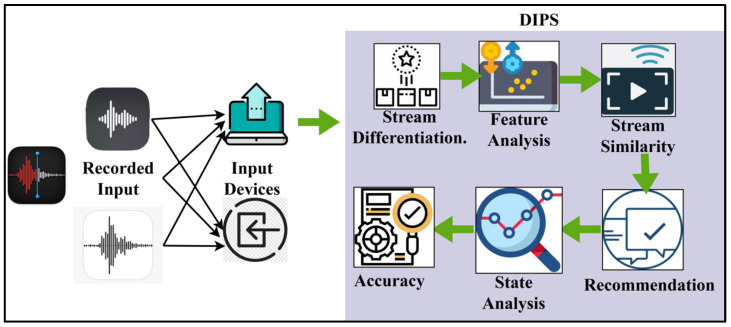
Discriminant Input Processing Scheme’s workflow.

Patients will receive self-assisted healthcare services and diagnoses based on observation with the help of Emotion-Aware Intelligent Systems designed for use in healthcare facilities. By relying on experience rather than cutting-edge technology, this system deviates from the norm in meeting user requests. Stream differentiation, feature analysis, stream similarity, suggestion, state analysis, accuracy, and recorded input from the input device are all supported by this system. All of the healthcare input devices share the recorded input. Several phases involve healthcare-related emotion classification from data acquired by input devices, including wearable sensors, before data stream differentiation for subsequent processing and analysis. Devices that receive signals relating to the user’s activity and physiological state collect raw data. Multifunctional sensors, audio devices, and visual gadgets might all fall under this category. These characteristics are vital to extracting relevant features from the preprocessed data to differentiate between various emotional states—features related to the body, sounds, and sight. Matching biological signals with their related aural and visual data is one example of synchronizing data streams; merging the characteristics gathered into a single dataset can help. The recorded input, stream distinction, and similarity primarily determine the emotion-aware healthcare system properties shared by DIPS. The workflow of the suggested method is shown in Figure 1.

There are several stages in DIPS that are used to capture and preprocess emotions. Data storage and retrieval, data accuracy and falsity analysis, approximation techniques to simplify the analysis, and data used to produce insights and predictions for individualized healthcare services are all part of the process. Emotional signals can be recorded using sensors or monitoring devices. This procedure improves the services and diagnoses provided by self-assistance healthcare providers.

DIPS stores and retrieves data from the input device’s recorded input. Accuracy, false data, approximation, analysis time, and data usage are all aspects of recorded input-based emotion data analysis that the suggested scheme adheres to. Q represents the recorded sequence input from the intelligent computing center’s observation-based diagnostic, where information is updated. The input device is investigated for patient emotion recording and data projection in a human-understandable format. The observation-based diagnostic center offers self-assisted healthcare services. The input device records these emoticons. The healthcare analysis monitors this recorded input using the input device. Assume that n stands for the patient’s healthcare data analysis. This system detects the patient’s physiological emotions utilizing the input device. Individual patient ID numbers are used to separate each n. Grouping the patient ID with the approach list is necessary for DIPS to provide care. The diagnostic center revealed that this method has few certified users. A certified user is a patient registered with the diagnostic facility and assigned space with this patient ID.

In healthcare services and self-assistance diagnosis, DIPS tracks an individual’s emotional state using physiological emotions. Physiological responses to these feelings can include shifts in hormone levels, heart rate, blood pressure, and other vital signs. The patient’s general health, stress levels, and emotional reactions can be better understood when medical personnel analyze and interpret these feelings. As a result of this integration, care and treatment plans for patients are more precise and efficient. Table 2 represents the notations and their definitions.

### 3.1. Observation-Based Diagnosis

The diagnostic center requires a computing model that is fair with DIPS. All of these computations are saved by the input device, which is controlled by the analysis time $A_{t}$. The DIPS service provider builds the use of stream differentiation $S_{d}$ and stream similarity $S_{s}$ features extracted for the segmented data $D_{s}$.

#### Preliminaries

With these segmented data $D_{s}$ for emotion analysis, the various streams converge using a futuristic process based on segmented data retained by the observation-based diagnostic center. The two stream computations $P_{c}$ operated depending on $A_{t}$ are derived as

(1) $$P_{c}=\{\prod_{j=1}^{i}\frac{j}{{\left(A_{t}\right)}_{i}}*\left[E^{k}\right],\;\in A_{t}>1\;\prod_{j=1}^{i-A_{t}}\frac{j}{\left[{\left(A_{t}\right)}_{i}-1\right]}*\left[E^{k}\right],\;\in A_{t}\leq 1\;$$

In Equation (1), i denotes the number of recorded inputs of n to be saved for starting the functions. Similarly, the recorded input and analysis time of observation-based diagnosis and DIPS are the same—i.e., $A_{t}>1$, otherwise, $A_{t}=\frac{A_{b}}{A_{c}}$; therefore, $A_{b}$ and $A_{c}$ are the recorded input and analysis, respectively. DIPS processes the recorded input from the input device. The first instance of recording input involves the process $A_{b}>$ $A_{c}$, and hence, the input device follows $P_{c}$ as per the sequence of the $A_{t}\leq 1$ condition. In a recording instance, the emotional data receive $E^{k}$ derived as

(2) $$E^{k}=\{\frac{x}{C_{p}}-\frac{A_{t}}{j},\;if\;0>A_{t}>1\;\frac{y}{C_{p}}-\frac{A_{t}}{\left(j-A_{t}\right)}\;,\;if\;0\leq A_{t}\leq 1\;$$

In Equation (2), x and y are the count of verified input sequences and varying emotion data sequences.

This occurrence of emotion sequence is recorded to $R_{i}$ of the observation-based diagnostic center. Therefore, the emotion data function is computed as

(3) $$n^{u}≅\|h\left(x,n\right)⊕Q\left[R_{i}\right]\|\left|x+j\right|\;and\;n^{v}≅\|h\left(y,n\right)⊕Q\left[R_{i}\right]\|\left|y+j-A_{t}\right|$$

In Equation (3), the variables $n^{u}$ and $n^{v}$ are the recorded emotional data for the input device and $n^{u}$ is the recorded input for the update. In this manner, both conditions of $\left[n^{u}S_{s}+xS_{d}\right]=\left[n^{v}S_{s}+xS_{d}\right]⊕\left[n^{u}S_{s}+yS_{d}-A_{t}\right]=\left[n^{v}S_{s}+yS_{d}-A_{t}\right]$ are achieved. Therefore, the emotion data n are said to be differentiated.

In Algorithm 1, to make it easier to understand, this version uses function calls or placeholders for the complex mathematical expressions and notations (such as calculate expression_1 and compute emotion data_1). Because of this, the complex complexities of the calculations are not as overwhelming, and the pseudocode is easier to read and comprehend. To make the pseudocode more concise, certain variables have had their names abbreviated, and unnecessary comments have been eliminated.

**Figure 2:**
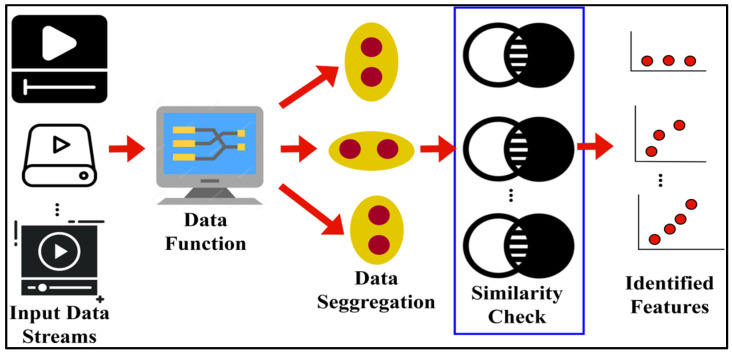
Feature-based analysis in DIPS.

The input device forms this intelligent computation of emotional data alongside the healthcare system approach list. The record contains the patient’s identification and the time of analysis. A patient’s $n^{u}$ and $n^{v}$ observations are encrypted using a specific performance. At this point, the detecting instance and patient ID are used to retrieve and determine the security portion of emotional data. This section will not be approved if the analysis duration is increased due to emotional data stored in DIPS or if it is accessed with a fraudulent communication patient ID. Figure 2 displays the feature-based diagnostic.
**Algorithm 1. Observation-Based Diagnosis*****Input:*** Initialize at initial analysis time $S_{d}$*:* Stream distinction feature $S_{s}$: Stream similarity feature $D_{s}$: Segmented data for emotion analysis $P_{c}$: Initial computation value ***Output:*** Compute: $P_{c}$ based on $A_{t}$, Adjust: $E^{k}$ based on $A_{t}$, Analyze: Emotion data function Swap: $P_{c}$ is based on feature-based analysis, and Deny: The access decision is based on $S_{d}$ and $S_{s}$ condition. ***Step 1:*** Compute $P_{c}$ based on $A_{t}$ ***if*** $A_{t}$ > 1 ***then***
***for*** j from 1 to I ***do*** $P_{c}$ += compute_expression_1(*j*, $A_{t}$, $E^{k}$) ***else*** ***for*** j from 1 to ($j-A_{t}$), ***do*** $P_{c}$ += compute_expression_2($j,\;A_{t}$, $E^{k}$) ***Step 2:*** Compute $E^{k}$ based on $A_{t}$ ***if*** $\;0\;>\;A_{t}$ > 1 ***then*** $E^{k}$ = compute_expression_3($x,\;C_{p}$, $A_{t}$, j) ***else if***
$\;0\;<=A_{t}$ <= 1 ***then*** $E^{k}$ = compute_expression_4($y,\;C_{p}$, $A_{t}$, j) ***Step 3:*** Compute the emotion data function $n^{u}$ = compute1(x, n, $R_{i}$, j) $n^{v}$ = compute2($y,\;n,\;R_{i}$, $j,\;A_{t}$) ***Step 4:*** Feature-based analysis ***if*** Ab! = Ac ***then*** Pc follows 0 $>=A_{t}$ >= 1 condition ***else if*** $A_{b}$$==A_{c}$$\;\mathrm{and}\;A_{t}$ == 1 ***then*** $P_{c}$ is swapped from x+y = 1 instance ***Step 5:*** Access SA SA based on SD and SA condition ***if*** SD > SA ***then*** Compute accuracy, false data, approximation, and data utilization ratio ***else*** Deny access to emotional data

It is not necessarily the case that additional characteristics or data points added to an ML model improve its accuracy. There are a few reasons why precision drops, such as overfitting and restricted generalizability, are consequences of the Curse of Dimension, which occurs when the amount of input characteristics sparsely grows at the expense of a feature. Not every feature helps make predictions. The model’s accuracy can be negatively affected by noise introduced by unnecessary or duplicated features. When there are more features to input, the model risks overfitting and capturing noise instead of the actual pattern in the training data. Unseen data perform poorly as a consequence. The computational expense for learning and forecasting goes up as the number of input features goes up, which might not be worth it if the reliability does not go up as well.

The data functions for segregation are responsible for inducing the channels of input data streaming. In this process, $\;A_{b}$ and $\;A_{c}$ are identified using the similarity check process. The similarity-verified data are agreed using the extracted features. In this feature extraction (as in Figure 2), limited ones are considered for verifying $0\geq A_{t}\geq 1$. As discussed above, if $\;A_{b}\neq A_{c}$, then $P_{c}$ follows the $0\geq A_{t}\geq 1$ condition. Alternatively, if $\;A_{b}=A_{c}$, and $\;A_{t}=1$, then $P_{c}$ is swapped from the x+y=1 instance such that $A_{t}$ is given in common by $P_{c}$ between the observation-based diagnostic center and DIPS. This transaction of arrangement currently functions in an incrementing sequence of x or y depending upon the $n^{u}$ and $n^{v}$ stream differentiation. The recorded input of the healthcare system offers stream differentiation based on x and y that is satisfied. Let $S_{D}$ represent the stream differentiation under a multiple-feature study such that emotion data analysis $S_{A}$ is analyzed. The emotion data analysis in the healthcare system is functioned from the input device for capturing the emotion and handling the sequence. The two conditions are used for classifying the data using features to the recommendation, i.e., either $S_{D}>S_{A}$ or $\;S_{D}\leq S_{A}$. These two conditions are analyzed in a varying instance to classify the emotion data in a better occurrence. The condition of $S_{D}>S_{A}$ is observed and establishes the extracted feature analysis given to the stream similarity function with respect to $P_{c}$ and $\;A_{t}$. Therefore, the condition of $S_{D}\leq S_{A}$ is not extracted by the feature analysis and needs the inaccessibility of emotion data, i.e., $P_{c}$ for $S_{D}>S_{A}$ and $S_{D}\leq S_{A}$ is defined as

(4) $${P_{c}}^{u}=\{\prod_{j=1}^{i}\frac{j}{{\left(A_{t}\right)}_{i}}+\left[A_{i}\times S_{A_{i}}\right],\;if\;S_{D}>S_{A}\;\prod_{j=1}^{i-A_{t}}\frac{j}{\left[{\left(A_{t}\right)}_{i}-1\right]}+\frac{A_{i}}{\left[{S_{D}}_{i}-S_{A_{i}}\right]},\;if\;S_{D}\leq S_{A}\;$$

In Equation (4), ${P_{c}}^{u}$ is the variable that represents feature analysis operated by the emotion data through a patient. From this condition, the approval access on its stream differentiation and similarity $\;A_{u}$, $\;D_{u}$ basis is operated at the analysis time $S_{A}$. The encrypted data about the patient do not modify by third person by accessing the healthcare information changes with $\;S_{A}$. The similarity checks for ${P_{c}}^{u}$ access on the $A_{u}$ and $D_{u}$ recommendation. The modified emotion data shared between the healthcare system and the patient are recommended if $\;S_{D}>S_{A}$; it makes certain accuracy, false data, approximation, and data utilization ratio access to n.

### 3.2. Similarity Checking

DIPS is able to make precise assessments and suggestions because it employs similarity to compare and match patterns in recorded emotional data. Patterns can be better identified, recommendations can be more accurate, and data can be more effectively used in healthcare analysis with the help of similarity checks. Emotional data analysis, recommendation making, and self-assisted healthcare service efficiency and accuracy are all greatly enhanced by this approach.

Similarity checking is carried out per specific recommendation, and information is readily available inside healthcare systems. The least cost in analysis time-based verification was required for this similarity check. In checking for similarity, the simultaneous factor serves as the first instance. This procedure is considered to be $\;P_{c}$. It is shared between the healthcare system and the observation-based diagnostic center. Let $\{D_{1},\;D_{2},\ldots ,D_{n}\}${*D*_1_, *D*_2_…D_n_} be the sequence of the emotion data recommendation from the observation-based diagnostic center and be generated by intelligent computing.

This instance of checking similarity in the healthcare system denotes either $n^{u}$ or $n^{v}$ such that the secured healthcare data are given as if $n^{u}+\left[A_{t}*Q\left[R_{i}\right]\right]-S_{d}\left[A_{t}*\left(x-j\right)\right]=n^{u}+A_{t}\left[Q\left[R_{i}\right]*\left(x-j\right)\right]-Q\left[R_{i}\right]\left[A_{t}*\left(x-j\right)\right]$ or $n^{v}+\left[\left(A_{t}*Q\left[R_{i}\right]\right)-S_{d}\left[A_{t}\times y\right]\right]=n^{v}+A_{t}\left[Q\left[R_{i}\right]\times y\right]$. The above representation corresponds to the recommendation of $n^{u}$ or $n^{v}$. Therefore, the RHS is coordinated with the healthcare data properties $Q\left[R_{i}\right]$ and $\;S_{d}$. Hence, similarity verification experiences a change in sequence if $\;S_{D}>S_{A}$. Otherwise, if the change in recommendation is accessed, then

(5) $$\begin{array}{c} n^{u}+\left[A_{t}*Q\left[R_{i}\right]\right]-C_{p}\left[A_{t}*\left(x-j-\frac{S_{D}}{A_{t}}\right)\right]=n^{u}+A_{t}\left[Q\left[R_{i}\right]*\left(x-j-\frac{S_{D}}{A_{t}}\right)\right]-Q\left[R_{i}\right]\\ \left[A_{t}*\left(x-j-\frac{S_{D}}{A_{t}}\right)\right]\;n^{v}+\left[\left(A_{t}*Q\left[R_{i}\right]\right)-S_{d}\left[A_{t}\times y+\frac{\left(x-j\right)}{A_{t}}\right]\right]=n^{v}+A_{t}\left[Q\left[R_{i}\right]\times y+\frac{A_{t}}{\left(S_{D}-S_{A}\right)}\right] \end{array}$$

According to the equation presented above, the similarity check and recommendation of the emotion data for the various instances of $D_{1}$ to $D_{n}$ are computed for intelligent computing. The verification of similarity is carried out in line with the approximation, and the data utilization ratio is determined by Equation (6) as follows:

(6) $$S_{D}≃\frac{\rho \left(A_{t}\right)\cdot Q\left[R_{i}\right]\;\rho \left(S_{D}\right)}{\prod_{j+1}^{i}\left[{S_{D}}_{i}-S_{A_{i}}\right]}*n^{u}\;S_{A}≃\frac{\rho \left(A_{t}\right)\cdot \;Q\left[R_{i}\right]\;\rho \left(S_{A}\right)\cdot P_{c}}{\prod_{j+1}^{i}\left[A_{t}\times xy+\frac{\left(x-j\right)}{A_{t}}\right]{\left\{\left[{\left(A_{t}\right)}_{i}-1\right]\times \rho \left(Q\left[R_{i}\right]\right)\right\}}_{j}}*n^{v}$$

Equation (6) computes the calculation of the recommendations observed in $S_{D}$ and $S_{A}$ for the current state $\rho \left(A_{t}\right)$ that is protected from the previous state of analysis. In the initial state of the recommendation process, the estimation of $\;S_{D}$ and $S_{A}$ initial and final states $\rho \left(S_{D}\right)$ and $\rho \left(S_{A}\right)$, the following inputs are for transfer learning. This process is induced to identify similar data shared between multiple origins of streams in the given input. The arrangement of instance handling of stream similarity is used to find the false data in innovative processing between A and $\;D\;\forall \;\rho \left(A_{t}\right)$. This transfer learning process is well defined in the following process.

### 3.3. Transfer Learning for State Analysis

**Figure 3:**
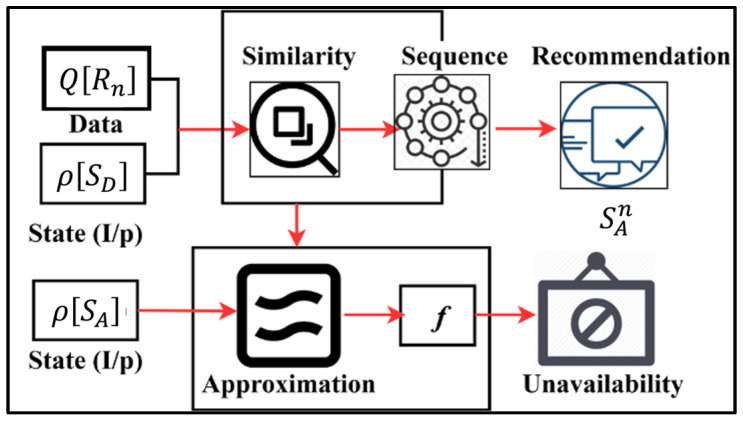
Transfer learning for recommendation state.

In the state analysis process, transfer learning is used to retain the previous recommendation  A or  D and data feature for further data utilization f in sequential data streams. This learning depends on previously saved recommendations from $A_{t}$; this ensures better state recommendation accuracy, regardless of the repeating recorded input data and similar characteristics, is achievable. The sequence of varying instances through intelligent computing helps to separate $A_{t}$ for both states of the intervals and $n\left(Q\left[R_{i}\right]\right)$ for all A. In this manner, the transfer learning process performs two types of stating: recommendation state and analysis state. In this state computation process, A and  D are required to increase the accuracy of emotion data $\;A_{t}$ in the healthcare system. Therefore, in the state analysis, the sequence of observed instance of $A_{t}$ is retained to improve the $Z_{t}$ join with better recommendation accuracy and detection of false emotion data. In particular, the sequence of emotion data differentiation is $A_{t}$ and $Q\left[R_{i}\right]$. Figure 3 presents the transfer learning process for the recommendation state.

The state input is fed for similarity and approximation measures in two instances. The proposed state verifies the approximation for identifying  f such that data unavailability is precisely determined. The computation is varied for $\;S_{A}^{n}$ and the similarity sequence for providing recommendations as output (Figure 3). The computation of $A_{t}\forall \;Q\left[R_{i}\right]\;$ is mapped under $S_{D}$ and $S_{A}$ depending upon the following instance of sequence. In this process, $A_{t}$ and the analysis time are categorized independently through state analysis.

Algorithm 2 defines function calls, comments, variables, and keywords well. The three primary operations are as follows: ‘TransferLearningForRecommendationState’ calculates the recommendation state sequence, ‘TransferLearningForStateAnalysis’ calculates the analysis state sequence, and ‘MainTransferLearning’ computes the final output by combining the two. Detailed mathematical calculations and formulae are not included in this simplified execution.
**Algorithm 2. Transfer Learning for State Analysis*****Input:*** data instance: data_instance_1, data_instance_2,…, data_instance_n ***Output:***
SD *SD*: recommendation state sequence; *SA*: analysis state sequence ***Step 1: function*** TransferLearningForRecommendationState(input_data) ***return*** // Compute recommendation state sequence ***for*** each data instance $\left(R_{i}\right)$ R_i in input_data, ***do*** SD[i] = compute_recommendation_state $\left(R_{i}\right)$ ***return***
*SD* ***Step 1: function*** TransferLearningForStateAnalysis(input_data) ***return*** // Compute analysis state sequence ***for*** each data instance $\left(R_{i}\right)$ in input_data, ***do*** SA[i] = compute_analysis_state $\left(R_{i}\right)$ ***return*** SA ***Step 2a:*** Update state analysis ***for*** each SA[i] in SA ***do*** ***if*** false_data_detected (SA[i]) ***then*** SA[i] = update_state_analysis (SA[i]) ***return*** SA ***Step 3: function*** MainTransferLearning(input_data) ***return*** SD = TransferLearningForRecommendationState(input_data) SA = TransferLearningForStateAnalysis(input_data) ***return***
SA, SD ***Step 4:*** Compute final output final_output = compute_final_output (SD, SA) ***return*** final_output

The state analysis processing is computed for $n\left(Q\left[R_{i}\right]\right)$ and $\left(n\left[R_{i}\right]\right)$ after which a precise transfer model helps to update the initial state. The state analysis sequence is estimated using Equation (7) as

(7) $$S_{D}=Q\left[R_{1}\right]\;{S_{D}}^{1}=2Q\left[R_{1}\right]+{\left(n\left[R_{i}\right]\right)}_{1}-\rho {\left(Q\left[R_{1}\right]\right)}_{1}\;{S_{D}}^{2}=3Q\left[R_{2}\right]+2{\left(n\left[R_{i}\right]\right)}_{2}-\rho {\left(Q\left[R_{2}\right]\right)}_{2}\;\vdots \;{S_{D}}^{n}=\;n\left(Q\left[R_{n}\right]\right)+n\left[R_{n}\right]-\rho {\left(Q\left[R_{1}\right]\right)}_{n+1}\;{︸}_{Recommendation\;state}|\;S_{A}=Q\left[R_{1}\right]\;{S_{A}}^{2}=2\left(n\left[R_{2}\right]\right)+\;\rho {\left(Q\left[R_{2}\right]\right)}_{2}\;{S_{A}}^{4}=4\left(n\left[R_{4}\right]\right)+\rho {\left(Q\left[R_{4}\right]\right)}_{2}-\rho {\left(Q\left[R_{4}\right]\right)}_{2}\;\vdots \;{S_{A}}^{n}=n\left[R_{n}\right]+\;\rho {\left(n\left(Q\left[R_{n}\right]\right)\right)}_{n+1}\rho {\left(n\left[R_{n}\right]\right)}_{n+2j}\;{︸}_{analysis\;state}\;$$

The estimation of state analysis generates two outputs, $Q\left[R_{n}\right]$ and $\left[R_{n}\right],$ from sequence ${S_{D}}^{1}$ to ${S_{D}}^{n}$ and updated instance ${S_{A}}^{2}$ to ${S_{A}}^{n}$, respectively. In particular, the mapping is performed under multiple streams based on the occurring sequence. In this case, the condition of $Q\left[R_{1}\right]\in S_{D}$ is not equal to $Q\left[R_{1}\right]\in S_{A}$ in the mapping condition. Therefore, if the state occurrence of ${S_{D}}^{1}$ is the initial state of emotion data, then ${S_{A}}^{2}$ is performed using $n\left[R_{n}\right];$ i.e., $Q\left[R_{1}\right]$ is divided as per the standard of $n\left[R_{n}\right]$ instance, and then $n\left[R_{n}\right]+\rho {\left(n\left(Q\left[R_{n}\right]\right)\right)}_{n+1}\rho {\left(n\left[R_{n}\right]\right)}_{n+2j}$ is the consecutive updating of the recommendation state instances. In this manner, the starting state is $\left(S_{D},Q\left[R_{n}\right]\right)$ from which $\left(S_{A},n\left[R_{n}\right]\right)$ is separated using segmented data. In these segmented data, the segmentation of $S_{D}$ and $S_{A}$ is derived such that $n\left[R_{n}\right]=\left\{n\left[R_{n}\right]\cup \rho \left(n\left[R_{n}\right]\right)\right\}$ and $Q\left[R_{n}\right]=\left\{S_{D}\cap \rho \left(n\left[R_{n}\right]\right)\right\}$ are mapped independently. The analysis state of the update sequence is achieved in its first state, from which the consecutive state is mapped alone. In both states, the false data are increased before the recommendation state is updated as

(8) $$S_{A}=\frac{1\left(n\left[R_{1}\right]\right)+Q\left[R_{n+1}\right]}{n\left[R_{1}\right]}\;{S_{A}}^{2}=\frac{2\left(n\left[R_{2}\right]\right)+Q\left[R_{n+2}\right]}{n\left[R_{2}\right]}-\rho \left(n\left[R_{n-1}\right]\right)\;{S_{A}}^{4}=\frac{3\left(n\left[R_{3}\right]\right)+Q\left[R_{n+3}\right]}{n\left[R_{3}\right]}-\;\rho \left(n\left[R_{n-2}\right]\right)\;\vdots \;{S_{A}}^{n}=\frac{j\left(n\left[R_{n}\right]\right)+Q\left[R_{n+j}\right]}{n\left[R_{n}\right]}-\rho \left(n\left[R_{n-j-1}\right]\right)$$

The state analysis update is estimated at segmented data of all $n\left[R_{n}\right]$ or the previous start of the next $\;Q\left[R_{n}\right]$. The state analysis update depends on ${S_{A}}^{n}$, $n^{u}$ being updated. The overall streams of the state analysis handle the state of $\left({S_{A}}^{n},n\left[R_{n}\right]\right)$ and $\;({S_{A}}^{n}$,$\;n^{u})$. In this process, the first ${S_{A}}^{n}$ denotes the update for $S_{D}$, and the next state denotes $\;S_{A}$. It is to be pointed out that either of the state ${S_{A}}^{n}$ that is mapped under $S_{D}$ or $S_{A}$ is updated with the emotion data in the state analysis. From the state analysis, the recorded inputs $\;n\left[R_{n}\right]$, $n^{u}$ and f are used for the consecutive assessment of the ${S_{D}}^{1}$ to ${S_{D}}^{n}$ sequence. In this condition, the false data are derived as

(9) $$f=\sum_{j+1}^{n}\frac{P_{c}A_{t}-\rho \left(Q\left[R_{n}\right]\right)*\left(n^{u}\right)+nA_{t}}{\left(P_{c}+n\right)t\;\rho \left(n\left[R_{n}\right]\right)+\rho \left(Q\left[R_{n}\right]\right)}+\frac{{\left(A_{t}\right)}_{i}}{\left[{\left(A_{t}\right)}_{i}-1\right]}$$

**Figure 4:**
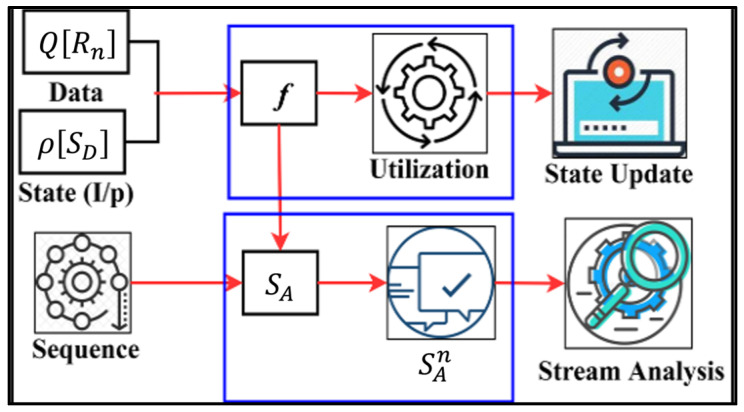
Transfer learning for state analysis.

As Equation (9) represents the variable, m is the occurrence of $n^{u}$ in $Q\left[R_{n}\right]$ as divided. This false data analysis helps us to compute the sequence of instance with the probability of mapping in either $n^{u}$ or $\;Q\left[R_{n}\right]$. Therefore, if  f=0, then the second instance if $\rho \left(Q\left[R_{n}\right]\right)$ is performed under $Q\left[R_{n}\right]$. Similarly, if $\;n^{u}<n^{v}$, then f=0 (i.e., the update sequence of f=0), which means that the sequential data streams of $\;n\left[R_{n}\right]$ are achieved. The accuracy of $n\left[R_{n}\right]$ is valid until the above condition fails. Hence, the consecutive occurrence of n sequentially varies both $\;n\left[R_{n}\right]$ and $Q\left[R_{n}\right]$ until the given state is discarded. Figure 4 presents the transfer learning process for state analysis.

Different from recommendation learning, the state analysis relies on the sequence and $\;S_{A}$ for analyzing the streams such that $\;S_{A}^{n}$ is identified under different utilization. The utilizations are distinguished between  f and state updates for preventing false data (Figure 4). A stream analysis is performed recurrently to improve accuracy if false data persist. The above process shows that self-assisted healthcare services and observation-based diagnosis for patients are derived through a sequence of estimations as per the following Equations (2), (3), (7), and (9) of either $n\left[R_{n}\right]$ and $Q\left[R_{n}\right]$ follows:

(10) $$n\left[R_{n}\right]=n^{u}*\rho \left(Q\left[R_{n}\right]\right)+S_{D}\;{\left(A_{t}\right)}_{i}\;R_{n}=\frac{j}{n^{u}}\left[n^{v}\rho \left(n\left[R_{n}\right]\right)+S_{D}\;{\left(A_{t}\right)}_{i}\right]\;$$

Equation (10) is the combined output of the state analysis and accuracy ${\left(A_{t}\right)}_{i}$ for all $n\left[R_{n}\right]$ instances. Hence, $Z_{t}$ in $Q\left[R_{n}\right]$ follows $\;\frac{j}{n^{u}}\left[n^{v}\rho \left(n\left[R_{n}\right]\right)+S_{D}\;{\left(A_{t}\right)}_{i}\right]$; this is shared between multiple streams of inputs f. Therefore, the data utilization ratio for the different patterns in emotion stream inputs for the instance of $Q\left[R_{n}\right]$ is derived as

(11) $$n\left(Q\left[R_{n}\right]\right)-\frac{j}{n^{u}}\;{\left(A_{t}\right)}_{i}=n\left[R_{n}\right]-n^{u}*\rho \left(Q\left[R_{n}\right]\right)-S_{D}f_{j}\;{\left(A_{t}\right)}_{i}+n\cdot n\left[R_{n}\right]\;\frac{j}{n^{u}}\;{\left(A_{t}\right)}_{i}=S_{D}f_{j}\;{\left(A_{t}\right)}_{i}+n^{u}*\;\rho \left(Q\left[R_{n}\right]\right)-n\cdot n\left[R_{n}\right]\;{\left(A_{t}\right)}_{i}=n\left[S_{D}f_{j}\;{\left(A_{t}\right)}_{i}+n^{u}*\rho \left(Q\left[R_{n}\right]\right)-n\cdot n\left[R_{n}\right]\right]\;$$

where the variable $S_{D}$ used in Equation (11) is computed from the consequences of $n\left[R_{n}\right]$ as in Equation (3), which is computed from $Q\left[R_{n}\right]$ as in Equation (5), derived as its sequences of recorded inputs. The emotional data analysis of healthcare systems, i.e., $Z_{t}$ with the false data f in $Q\left[R_{n}\right]$ in $n\left[S_{D}f_{j}\;{\left(A_{t}\right)}_{i}+n^{u}*\rho \left(Q\left[R_{n}\right]\right)-n\cdot n\left[R_{n}\right]\right]$, is the final estimation of $A_{t}$. If f and $S_{A}$ are not performed consecutively, then the entire state of $Z_{t}$ will be mapped under $f\left(Q\left[R_{n}\right]\right)$, resulting in high accuracy.

**Figure 5:**
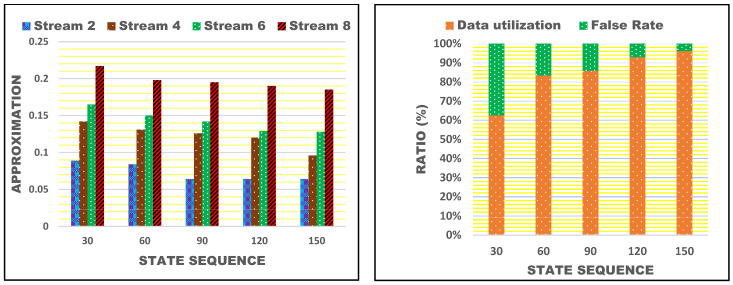
Approximation and ratio (data utilization and false rate) for different state sequences.

Figure 5 presents an analysis of approximation and ratio under different state sequences. The proposed scheme achieves less approximation by detecting $\;S_{A}<S_{D}$ and $\;S_{A}\geq S_{D}$ conditions and f reduction. This process is augmented using multiple stream analysis, reducing approximation. In the state-sequence-based data utilization, the change in $\;S_{A}^{n}$ and $\;S_{D}^{n}$ reduces f through the previous state update. $S_{A}^{n}$ and $\;S_{D}^{n}$ are utilized for different computing instances, maximizing data utilization. Therefore, as data utilization increases, the false rate decreases based on $n\left[R_{n}\right]$ and $n\left(Q\left[R_{n}\right]\right)$ sequences. Table 3 presents the similarity ratio for different data input streams.

Table 3 analyzes the similarity ratio for different data input streams. The proposed scheme improves feature extraction by maximizing $\;E^{k}$ exploitation. In the learning recommendation, the variations are reduced by preventing  f in $\;n\left[R_{n}\right]$ and $\;S_{A}^{n}$-based updates. This achieves fair data utilization regardless of the feature extraction ratio, which reduces f and maximizes data utilization and similarity. In Table 4, the false rate for different inputs is presented.

Table 4 displays the input streams (120) derived from the patients’ recorded inputs using the emotion data analysis and individual patient ID numbers. According to the diagnostic center, only a few certified users use this procedure. Patients who signed up with the diagnostic center and received a space assignment using this patient ID are considered certified users.

Table 4 presents the values for false rates for different inputs. The change in state sequences is defined as high/low such that the high state sequences are experienced if unavailability is high. Contrarily, the unavailability results in a false rate. Therefore, the f mitigation is prevented based on availability, avoiding an increase in false rate.

## 4. Results and Discussion

### 4.1. Dataset Description

This section continues by presenting the performance analysis through a comparative discussion. The ascertained dataset [32] is utilized when evaluating and distinguishing various emotions. This dataset gives emotional data based on physiological responses recorded using EEG, ECG, and facial expression labeling. This research introduces ASCERTAIN, a database that uses commercial physiological sensors to provide multimodal information for implicit personality and affect recognition. Using commercially available sensors, ASCERTAIN records the EEG, ECG, GSR, and facial activity of 58 users in real-time as they watch emotionally charged movie clips, in addition to their Big Five personality traits and emotional self-ratings. Our investigation begins with a review of the literature on users’ emotional assessments and personality measures, followed by a review of the linear and nonlinear physiological correlates of temperament and emotion. The experimental results show that comparing user reactions with emotionally homogeneous movies is the best way to uncover personality differences. Both the affective and personality dimensions can achieve above-chance recognition. Inputs are collected from 50 individuals utilizing five personality traits with 180 labels. Raw analysis is performed on the video data for emotion identification for 20 s. The average age of the 58 participants was 30 years, and they were exposed to 36 movie clips in the study. Electrocardiograms, galvanic skin responses, electroencephalography, and trajectories of face landmarks were used to synchronize the data. Annotations regarding the quality of all recorded data, Big Five personality trait scores, ratings of 50 descriptive adjectives, and self-reports for 36 videos, including 58 individuals, were all part of the data review. Examining how viewing videos affects character quirks was the primary goal of the research. Accuracy, false rate, approximation, analysis time, and data consumption parameters are all subject to the comparative analysis. This study compares the proposed scheme with existing studies, namely, Valence Lateralization with Ensemble Learning (VL+EL) [27], Fractal Pattern Feature-Based Emotion Recognition Approach (FPFEA) [20], and MSD [19].

### 4.2. Accuracy Comparison

**Figure 6:**
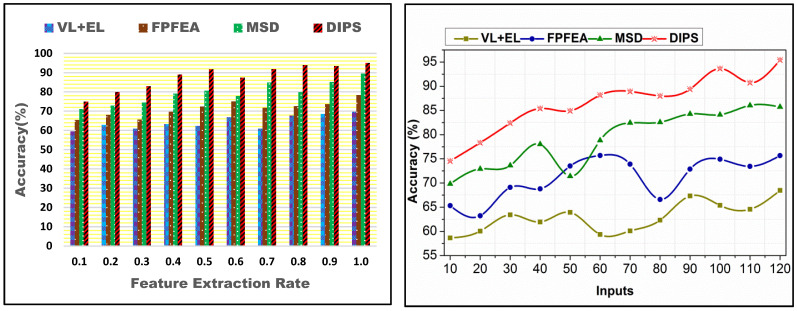
Accuracy comparisons.

The proposed scheme achieves fair accuracy for different feature extraction rates and inputs. In this scheme, similarity and $\;S_{A}$ verification achieve the detection and analysis without errors. $A_{b}\neq A_{c}$ and $\;S_{D}\leq S_{A}$ analyze the input for $\;P_{c}^{u}$ identification. This identification reduces the input (false) processing, improving $\;n^{u}$. The state sequences are validated using $\;S_{A}^{n}$ by assigning different iterative computations. In the different input $\;S_{D}$, the feature extraction is maximized by providing  f in different $\;R_{n}$. Based on the features,$\;S_{D}^{n}$ and $\;S_{A}^{n}$ are detected, and $S_{D}^{n}$ is capable of providing different outputs for the requesting users through services. This alone increases the accuracy. However, the analysis state continues to mitigate to maximize accuracy. The recurrence in transfer state learning maximizes $n\left(Q\left[R_{n}\right]\right)$ by reassigning the state sequences. Finally, the $Z_{t}$ mapping for $\;f\left(Q\left[R_{n}\right]\right)$ maximizes accuracy for the consenting $\;n\left[R_{n}\right]$. Therefore, the accuracy is precisely high, as presented in Figure 6.

Adding traits or data points to a machine learning model’s input does not necessarily make it more accurate. Standard methods for dealing with an abundance of input features, particularly when it surpasses the number of training examples, include regularization, dimensionality reduction, and feature selection. When training a model, it is critical to balance the amount and quality of the characteristics and information points used as inputs. The suggested model uses feature analysis, which means that the model’s accuracy in Figure 6 will remain unaffected by the increased output.

### 4.3. False Rate Comparison

**Figure 7:**
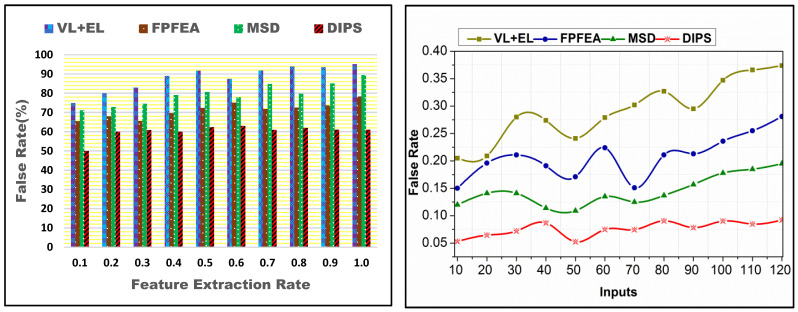
False rate comparisons.

A comparative analysis for different feature extraction rates and inputs is presented in Figure 7. The $S_{A}$ and $\;S_{D}$ processes are validated for identifying  f such that the sequences are analyzed as $\frac{A_{t}}{\left(S_{D}-S_{A}\right)}$. In transfer learning, $\rho \left(n\left[R_{n}\right]\right)$ is analyzed such that the instance $Q\;\left[R_{1}\right]$ is segregated for  (n+1) and  (n+2j)∀ j∈Q. This segregation is performed for identifying $\;nA_{t}$ and $\;\rho \left(n\left[R_{n}\right]\right)$. In this identification, $\;n^{u}$ occurrence in $\;Q\left[R_{n}\right]$ alone generates  f that is mitigated using recurrent analysis until the false state is reduced. It is extended for the sequences $\;n\left[R_{n}\right]$ and $\;R_{n}$ such that  f in any $\;R_{n}$ is reduced under different processing instances. The analysis is carried out until the accuracy (maximum) is reached. Therefore, $\frac{j}{n^{u}}\;\forall f\in R_{n}$ is used alone for computing the accuracy in identifying  j. It is unanimous for different inputs based on the extracted features.

### 4.4. Approximation Comparison

**Figure 8:**
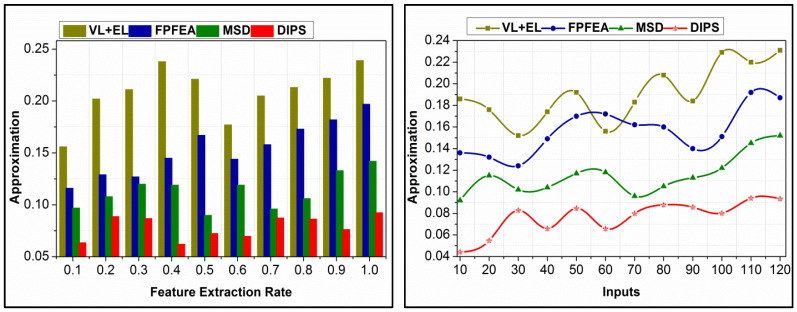
Approximation comparisons.

The proposed scheme identifies f-based approximation for maximizing accuracy. The proposed $\;P_{c}$ is verified based on similarity verification such that $\;S_{A}$ is improved. The state sequences are analyzed based on $\;S_{D}^{n}$ and $\;S_{A}^{n}$ for $n\left(Q\left[R_{n}\right]\right)$. Based on $\;n^{v}$ and $\;n^{u}$, the analysis for approximation requiring and leaving out sequences are identified. Through this identification, the learning and training iterates are classified. The sequence is determined for $\;\left[A_{t}*\left(x-j\right)\right]=n^{u}$ such that different approximation levels are retained. This is required for validating the data sequence provided that $Q\left[R_{i}\right]\forall i\in n$ and $\;S_{d}$ are matched. Contrarily, if $\;S_{D}>S_{A}$ is achieved, then $\;\frac{\left(x-j\right)}{A_{t}\;}\;\forall \;j\in S_{D}$ is approximated independently to satisfy either $\;S_{d}>S_{A}$ or $\;S_{D}\leq S_{A}$. Therefore, the approximation requirements are provided for different $\;n^{v}$ and $\;n^{u}$ requisites. $\rho \left(A_{t}\right)$ is updated as $\;S_{A}^{2}$ to $S_{A}^{n}$ through different training iterations, reducing the approximation (refer to Figure 8).

### 4.5. Analysis Time Comparison

**Figure 9:**
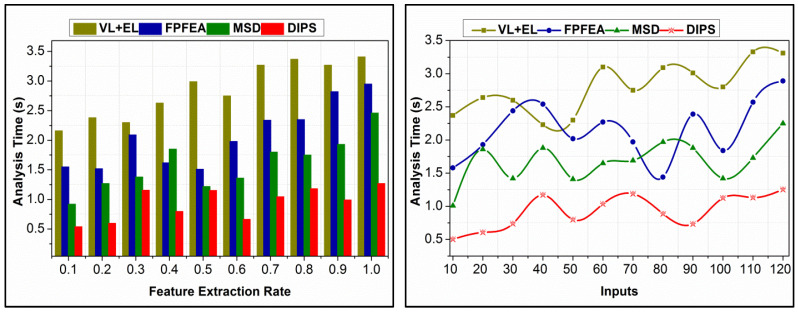
Analysis time comparisons.

For the different feature extraction rates and inputs, the analysis time is presented in Figure 9. In the proposed scheme, the analysis is segregated for  f−based and f−confined instances. In the learning-aided process, $\;S_{D}$ and $\;S_{A}$ determine the utilization at a maximum level. The proposed scheme recommends the assigned data without requiring additional instances. $\rho \left(Q\left[R_{i}\right]\right)\forall \;i\in n$ determines the computation requiring instances, preventing losses in augmentations of $\;A_{t}$, preventing additional time. In the similarity-checking process, $\;n\left[R_{n}\right]$ and $\;\left(S_{A}^{n},\;n\left[R_{n}\right]\right)$ are segregated for independent analysis. Based on this process, the computations are distinguished without requiring additional time. The sequence $\;n\left[R_{n}\right]$ and $\;R_{n}$ are identified independently without maximizing the computation time. Therefore, different processes in data analysis are preserved without confining $\;S_{D}>S_{A}$ such that the time requirements are smaller. The process varies with the inputs associated with it and the features extracted to retain unanimous results throughout.

### 4.6. Data Utilization Comparison

**Figure 10:**
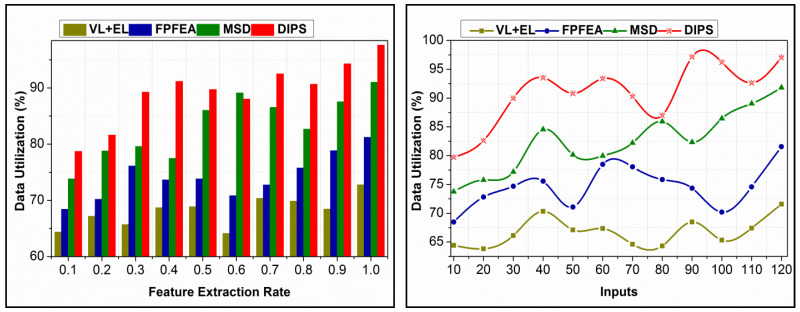
Data utilization comparisons.

A comparative analysis of data utilization for different feature extraction rates and inputs is given in Figure 10. The inputs are utilized for accuracy maximization and  f reduction based on transfer learning recommendations. $n\left[R_{n}\right]$ and $\;\left[R_{n}\right]$ determine the data requirements for further detection, provided that approximation and f are reduced. In the pursuit processes, $P_{c}^{u}$ is reduced by identifying different features recurrently. This identification defines $\;S_{D}^{n}$ and $\;S_{A}^{n}$ such that $\;n\left(Q\left[R_{n}\right]\right)$ is maximized as $\forall \;S_{D}^{n}$ in the first training phase. Contrarily, the following $\;S_{A}^{n}$ is analyzed for  f, from which $\;S_{A}$-preceded data are used for computation. The incoming $\;P_{c}$ is defined using multi-$E^{k}$ such that $\;S_{A}^{n\;}$ is performed for $n\left(Q\left[R_{n}\right]\right)$ in different  i∈n. Therefore, the utilization in the first instance greatly prevents further requirements. As the feature extraction rate increases, the utilization is pursued in achieving the maximum $\;E^{K}$ exploitation. This is included for all $\;S_{D}$ observed in different analysis times, requiring computational instances. These instances are responsible for maximizing data utilization. Table 5 presents the false rate and accuracy for different state sequences for five different emotions.

For the emotions above, first, we examine the collected data; among them, “happy” or “smiling” frequently yields accurate results. Second, the data make it easy to identify mood changes, while the others are categorized as miscellaneous. The contrasts mentioned earlier are presented in Table 6 and Table 7.

Summary: The proposed DIPS maximizes accuracy and data utilization by 15.9% and 15.96%, respectively. It reduces false rate by 8.75%, approximation by 10.02%, and analysis time by 9.47%.

Summary: The proposed scheme achieves 18.8% high accuracy, 9.56% low false rate, 9.65% low approximation, 9.24% low analysis time, and 15.42% high data utilization.

According to the diagnostic facility, few certified users use this process. Accredited patients are diagnostic center sign-ups who acquired a space assignment using this patient ID. In Figure 10, the data points are not very similar; there is a difference in patients’ emotions, so the false rate, accuracy, and other metrics vary in data points. Machine learning models may not be more accurate with more attributes or data. When input features exceed training examples, regularization, dimensionality reduction, and feature selection are used. The volume and quality of input characteristics and information points must be balanced when training a model. Since the suggested model uses feature analysis, the additional output will not impair accuracy. The computational cost of learning and forecasting increases with the quantity of input features, which could be detrimental if the dependability does not improve simultaneously.

## 5. Conclusions and Future Study

DIPS makes integrating emotion analysis into self-assisted intelligent healthcare systems possible, which is encouraging. Input devices can save and retrieve emotional data, improving healthcare services’ precision and efficiency. Patient care and treatment outcomes can be improved by better understanding patients’ well-being and emotional responses through the system’s focus on physiological emotions. Limited certified users, skewed or inadequate data, inadequate computational resources, and the inability to generalize across various healthcare settings and demographics are some of the difficulties that DIPS encounters. Research into DIPS’s potential uses in certain healthcare domains or populations, optimization of computing efficiency, enhancement of data quality, and ongoing development to guarantee its utility and robustness in intelligent healthcare systems should all be priorities for future studies. These are the areas where DIPS can improve and become a more helpful tool for AI healthcare systems sensitive to patients’ emotions.

## Figures and Tables

**Table 1:** Related works.

Author	Proposed Method	Application Used	Outcomes	Limitations
Meng et al. [17]	Emotion-aware healthcare monitoring system	Internet of Medical Things, EEG	High efficiency and accuracy of emotions	Scalability and generalizability of the hybrid emotion-aware monitoring system, especially for diverse patient populations and real-world implementation
Dhote et al. [18]	Mobile healthcare apps using distributed cloud technologies	Distributed Data Analytics and Organization Model, federated learning	Effective service rollout, improved data organization	Early service and recommendation problems
Li et al. [19]	Multistep deep (MSD) emotion detection system	Deep learning, imputation method	Improved performance by eliminating invalid data	Generalization of the multistep deep system across different IoT environments and additional validation of emotion detection under diverse conditions
Tuncer et al. [20]	Automatic emotion recognition system using EEG	EEG, facial patterns, iterative selector	High accuracy in emotion classification	The fractal pattern feature generation method’s scalability and robustness across different EEG data sources and neurological conditions
Ahamed [21]	Smart ageing with fall detection and dementia diagnosis	Biometric security, fall detection, dementia diagnosis	99% accuracy in fall detection, 93% accuracy in dementia diagnosis	Generalizability and effectiveness of innovative ageing solutions across diverse contexts and further validation of machine learning algorithms
Fei et al. [22]	Emotion analysis framework using Deep CNN	Analysis of the emotions of patients in healthcare	Increased accuracy in predicting emotions	Additional validation of the emotion analysis framework in clinical settings and its performance across demographic groups
Du et al. [23]	Hydrogel-based wearable and implantable devices	Hydrogel structure, piezoelectric capabilities	Flexible and stretchable devices, biomedical applications	Translating hydrogel-based piezoelectric devices to practical biomedical applications, including challenges related to stability and biocompatibility
Subasi et al. [24]	EEG-based emotion recognition with noise reduction	Discrete Wavelet Transforms, tunable Q wavelet transform	Maximized classification accuracy	Generalization of EEG-based emotion recognition to real-world scenarios and diverse datasets
Kao et al. [25]	Piezoelectric and triboelectric nanogenerators for wound healing	Piezoelectric and triboelectric materials	Potential use in wound healing, external electric field	Challenges in implementing self-assisted wound healing using nanogenerators, including biocompatibility and device performance
Dheeraj et al. [26]	Text-based emotion recognition using multi-head attention and BCNN	Multi-head attention, bidirectional convolutional neural network	Identification of negative next-based emotions, examination of mental-health-related questions	Generalizability of the deep learning model for negative emotion detection in mental-health-related texts, including biases in training data
Pane et al. [27]	Ensemble learning and lateralization approach	EEG-based emotion recognition, hybrid feature extraction, random forest	Improved emotion recognition accuracy	Generalization of the EEG emotion recognition method to diverse datasets and emotion categories
Anjum et al. [28]	Behavior-based response model for smart city traffic	Regression model, cloud computing	Real-time insights for drivers, congestion reduction	Scalability and real-world applicability of the behavior-based response model for traffic monitoring, including data privacy challenges
Upreti et al. [29]	Cloud-based model for smart city traffic analysis	Cloud computing, regression model	Real-time insights for drivers, traffic assistance	Generalizability of the IoT-assisted healthcare monitoring system to diverse settings and need for further validation in clinical environments

**Table 2:** Notation and definitions.

Notation	Definition
$A_{t}$ *A*_*t*_	Recorded input and analysis time
$A_{b}$	Recorded input from the input device
$A_{c}$	Analysis of the recorded input
$E^{k}$	Emotion data received
x	Count of the verified input sequence
y	Varying emotion data sequence
$P_{c}$	Process condition
$S_{A}$	Stream similarity
$S_{D}$	Stream distinction
$S_{A_{n}}$	State analysis
$S_{D_{n}}$	Feature extraction
f	Feature extraction rate
n	Iterative computations
Q	Recorded sequence input
$Z_{t}$	Mapping for maximizing accuracy
$n^{u}$ *n* ^*u*^	Input processing improvement
$n\left[R_{n}\right]$	Requesting users through services
$f\left(Q\left[R_{n}\right]\right)$	Feature-based accuracy maximization

**Table 3:** Similarity ratio for different streams.

Streams	Feature Extraction	Data Utilization (%)	Similarity (%)
1	0.16	63.5	55.82
2	0.25	71.69	63.25
3	0.31	68.25	59.87
4	0.38	75.36	74.25
5	0.42	73.98	68.25
6	0.39	82.69	78.21
7	0.58	89.54	81.36
8	0.69	92.64	89.25
9	0.97	97.57	90.81

**Table 4:** False rate for different inputs.

Inputs	State Sequences	Unavailability	False Rate
20	39	0.073	0.04
40	96	0.096	0.08
80	128	0.15	0.21
120	153	0.22	0.38

**Table 5:** False rate and accuracy for different state sequences.

Emotion	State Sequence							
	40		80		120		160	
	Accuracy	False Rate	Accuracy	False Rate	Accuracy	False Rate	Accuracy	False Rate
Anger	59.32	0.08	63.41	0.071	71.4	0.065	86.5	0.061
Sad/Crying	61.3	0.07	66.47	0.062	70.06	0.06	77.5	0.052
Happy/Smiling	67.3	0.063	74.6	0.059	78.2	0.056	90.07	0.054
Mood Change	81.3	0.043	86.51	0.039	90.39	0.039	94.19	0.035
Miscellaneous	80.4	0.058	83.62	0.051	86.1	0.048	90.39	0.048

**Table 6:** Comparison for feature extraction rate.

Metrics	VL + EL	FPFEA	MSD	DIPS
Accuracy (%)	69.68	78.34	89.47	95.059
False Rate	0.365	0.255	0.158	0.0843
Approximation	0.239	0.197	0.142	0.0925
Analysis Time (s)	3.41	2.95	2.46	1.269
Data Utilization (%)	72.78	81.23	91.01	97.624

**Table 7:** Comparison for inputs.

Metrics	VL + EL	FPFEA	MSD	DIPS
Accuracy (%)	68.48	75.67	85.74	95.429
False Rate	0.374	0.281	0.195	0.0921
Approximation	0.231	0.187	0.152	0.0935
Analysis Time (s)	3.31	2.89	2.25	1.254
Data Utilization (%)	71.58	81.57	91.81	97.072

## Data Availability

The data that support the findings of this study are openly accessible at the following link: https://sensor.informatik.uni-mannheim.de/#dataset_realworld_subject7, accessed on 5 March 2024.
